# Controversies on autoimmunity and prognosis in cancer

**DOI:** 10.1038/sj.bjc.6603313

**Published:** 2006-08-22

**Authors:** G Ferretti, A Felici, F Cognetti

**Affiliations:** 1 Division of Medical Oncology A, Regina Elena Cancer Institute, Rome, Italy

**Sir**,

The presence of CD4+CD25+ regulatory T cells (Treg) in cancer patients might be, in part, responsible for downregulation of antitumour immune responses. Schaefer *et al* (2005) have shown the importance of Treg in defining the immune profile of cancer patients, illustrating the role of these cells in downregulating functions of other T-cell subsets. Their hypothesis was that lymphocyte homeostasis disrupted by the presence of tumour fails to normalise following successful therapy. Wei *et al* (2005) recently assessed the possible synergy of immune reactivity to defined tumour- and self-antigens following reduction of CD4+CD25+ Treg. In a Treg-deprived environment, tumour cells primed the immune system, with consequent tumour regression. Treg-depletion enhanced autoimmunity to mouse thyroglobulin (mTg) in resistant BALB/c mice. Concurrent tumour regression and mTg immunisation resulted in further elevation of both antitumour and anti-mTg immunity.

There are various inhibitory mechanisms in T cells to prevent pathological autoimmunity. Blocking CTLA4 on T cells or depleting CD25+ Treg is sufficient to break self tolerance, causing nonspecific autoimmune pathologies (Phan *et al*, 2003). On the other hand, an association between autoimmunity and a favourable antitumour effect has been reported. Anti-CTLA-4 Ab given to patients with metastatic melanoma induces durable objective clinical responses associated with the induction of autoimmune side effects. The autoimmune and antitumour effects seen after CTLA-4 blockade appear to act through direct activation of CD4+ and CD8+ effector cells. In addition, inhibition of a signaling inhibitor in antigen-loaded dendritic cells (DCs) can induce a tumour-associated antigen-specific pathological autoimmune response against tumour. SOCS1-silenced DCs effectively break tolerance at the host level and cause self antigen-specific autoimmune pathologies against normal tissues but also against tumours (Evel-Kabler *et al*, 2006).

As interferon alfa-2b has the ability to induce an immune response against autoantigens with serologic and clinical manifestations of autoimmunity, it is possible that it also induces an immune response against tumours. The appearance of autoantibodies or clinical manifestations of autoimmunity during treatment with high-dose adjuvant interferon alfa-2b was associated with statistically significant improvements in relapse-free survival and overall survival (Gogas *et al*, 2006).

By contrast, the early presence of autoantibodies in the serum has been described to correlate with an unfavourable prognosis (de Visser *et al*, 2006). The occurrence of autoantibodies in the serum of cancer patients, and interstitial antibody deposition in human tumours have been widely reported. As CD4+ and CD8+ T-lymphocytes are important modulators of tissue-damaging B-lymphocyte responses, considered that Ig deposition occurs in human premalignant and malignant tissues, imbalanced adaptive-immune-cell interactions could represent underlying mechanisms that regulate the onset of chronic inflammation associated with cancer development (de Visser *et al*, 2006).

While it could be possible to use autoimmune responses as surrogate markers to evaluate new treatments (Gogas *et al*, 2006), it might be highlighted that individuals with progressive tumours could produce a higher antigen load, therefore triggering greater antibody deposition (de Visser *et al*, 2006).
